# Total neoadjuvant therapy followed by total mesorectal excision for rectal cancer in older patients real world data and proof of concept

**DOI:** 10.3389/fsurg.2024.1448073

**Published:** 2024-11-19

**Authors:** Isacco Montroni, Francesca Di Candido, Giovanni Taffurelli, Stefano Tamberi, Elisa Grassi, Jody Corbelli, Floranna Mauro, Enrico Raggi, Anna Garutti, Giampaolo Ugolini

**Affiliations:** ^1^ General Surgery Unit, Ospedale Santa Maria Delle Croci - AUSL Romagna, Ravenna, Italy; ^2^ General Surgery Unit, Ospedale per gli Infermi - AUSL Romagna, Faenza, Italy; ^3^ Medical Oncology Unit, Ospedale per gli Infermi - AUSL Romagna, Faenza, Italy; ^4^ Medical Oncology Unit, Ospedale Santa Maria Delle Croci - AUSL Romagna, Ravenna, Italy; ^5^ Department of Radiation Oncology, Maria Cecilia Hospital, Cotignola, Italy; ^6^ Department of Medical and Surgical Sciences, University of Bologna, Bologna, Italy

**Keywords:** rectal cancer, elderly, neoadjuvant therapy, transanal total mesorectal excision, functional recovery

## Abstract

**Background:**

Rectal cancer (RC) commonly affects older patients. Total Neoadjuvant Therapy (TNT) has been introduced to improve local and systemic control of RC. The aim was to present real-world data of older patients receiving TNT followed by surgery after a frailty assessment and verify feasibility and safety of this approach.

**Methods:**

This was a single-center retrospective study which enrolled all patients ≥70 years of age with RC who underwent TNT followed by surgery between November 2017 and April 2022. Data regarding cancer characteristics, neoadjuvant chemoradiotherapy (CRT), and toxicity were recorded. All patients underwent surgery 12–16 weeks after the end of therapy. Intra- and postoperative outcomes were recorded. Pre- and postoperative functional evaluation was carried out.

**Results:**

Fifteen patients were enrolled. Mean age was 74 (70–81) years. Mean distance of the tumor from the anal verge was 5.2 cm. Fourteen patients had positive nodes (93.3%), 11 (73.3%) showed involvement of the circumferential margin (CRM+) and 10 (66.6%) had extramural vascular invasion (EMVI+). Ten patients (66.6%) received mFOLFOX-6 and 5 CAPOX (33.3%) followed by CRT. After CRT, positive nodes were reported in 4 cases (26.6%), CRM+ in 4 (26.6%), and EMVI+ in 1 (6.6%). Transanal total mesorectal excision (taTME) was performed in all cases. Median operative time was 280 min (110–420). Median length of stay was 4 days (3–29). One Clavien-Dindo grade 4 complication, no readmissions, and no variations in pre- and postoperative functional status within 30 days from surgery were reported. No positive distal or CRMs were detected. Three pathologic complete responses were reported (20%).

**Conclusions:**

TNT followed by TME is feasible and safe in older patients, with good clinical and oncologic outcomes. Patient evaluation is crucial for maximizing cancer care in fit older patients.

## Introduction

Colorectal cancer (CRC) is the third most diagnosed cancer in the world and the second most deadly, with approximately 2 million new cases and 1 million deaths reported in 2018 (1, 2). Rectal cancer (RC) represents a third of all CRC cases and commonly affects older people. The median age at the time of diagnosis is 70 years, and relative incidence increases with age, having a peak incidence at 80–84 years (3). Due to the progressive aging of the population, the overall number of older patients with RC is expected to increase even more. Unfortunately, older patients are often undertreated or overtreated based on their chronological age alone (4, 5), generating a significant disparity as compared with younger patients (6). Chronological age remains one of the most used variables to assess outcomes of several medical and surgical treatments. Due to the increasing of life expectancy over time, older age reference points of 70 and 75 years have been used (4, 7, 8). Older adults are a heterogeneous population that covers a spectrum from frail to fit patients; however, treatment continues to be insufficiently tailored to each patient's specific needs. There is a paucity of trials addressing the risks and benefits of all aspects of RC care (9).

The standard treatment of RC is challenging for older patients as it often involves neoadjuvant chemoradiation therapy (as in the case of locally advanced RC), extended surgery (often involving a temporary or permanent stoma creation) and then more adjuvant care. In addition, new regimens based on extended preoperative chemotherapy treatment have been introduced into guidelines under the term Total Neoadjuvant Therapy (TNT) (10, 11). The thinking behind more intense preoperative management is to improve local and systemic control in high-risk patients showing threatened circumferential resection margins (CRM+s), the presence of extramural vascular invasion (EMVI+) and metastatic mesorectal and extramesorectal lymph nodes (12–14). The results of this new regimen seem to be extremely encouraging, and many centers around the world have incorporated TNT into their multidisciplinary treatment strategy (15–17). Unfortunately, to date, none of the randomized control trials testing TNT have been designed for older patients who have again been left out of the innovation process, only then to often be the recipients of these treatments in the real world. At the same time, if TNT is going to show sustained oncologic benefits, as it currently seems, prejudice and misjudgment towards older patients may prevent many people from receiving optimal care, only based on chronological age or perceived, non-assessed vulnerability.

The aim of this study was to present real-world data regarding older patients receiving TNT followed by minimally invasive Total Mesorectal Excision (TME) after a multidimensional frailty assessment. The primary goal is to verify whether this approach would be feasible and safe, and the secondary goal of this study is to show possible short-term oncologic outcomes improvements.

## Methods

### Patient characteristics

This was a single-center retrospective study based on a prospectively maintained database that enrolled all patients ≥70 years of age with biopsy-proven locally advanced RC (LARC) who underwent TNT followed by surgery between November 2017 and April 2022. Preoperative staging included colonoscopy, magnetic resonance imaging (MRI) and computed tomography (CT) according to the 8th edition of the American Joint Commission on Cancer (AJCC) staging system (18). All patients received initial frailty screening including the Charlson Comorbidity Index (CCI), Flemish Triage Risk (fTRST), Activities of Daily Living (ADL), Eastern Cooperative Oncology Group Performance Status (ECOG-PS) and nutritional screening. All the cases were discussed at diagnosis, after completion of the staging, and after treatment and re-staging by a multidisciplinary team (MDT).

The local MDT included colorectal surgeons, oncologists, radiotherapists, radiologists, pathologists, gastroenterologists, and geriatricians. The study was conducted in accordance with the latest revision of the Declaration of Helsinki and approved by the local Institutional Review Board (Comitato Etico della Romagna, C.E.ROM.). Informed consent was obtained from all subjects and/or their legal guardian(s).

Data regarding the tumor-node-metastasis (TNM) stage, the distance of the tumor from the anal verge, the total number of neoadjuvant chemo-radiotherapy (nCRT) cycles received, any toxicity during treatment and the need for dose reduction were prospectively recorded. All the patients underwent surgery 12–16 weeks after the end of radiation therapy. No watch-and-wait strategy was offered to patients undergoing TNT based on the insufficient knowledge of the long-term results of these patients by the time the protocol was adopted (November 2017). It was always made clear that the decision to undergo a TNT vs. standard chemoradiation was based on the goal of possibly obtaining improved long-term oncological outcomes rather than achieving a complete clinical complete response or pursuing sphincter-saving surgery.

Surgical options were collegially discussed first, during MDT, and with the patients and families then. The main criteria to offer a restorative vs. a non-restorative procedure (permanent colostomy) were based on oncologic criteria (invasion or threatened sphincter complex); patients' pre-existing clinical conditions (fecal incontinence, lack of independence in the self-care); patients' and family preferences. The contact of a patients' advocate/witness (mainly former patients in the same age group who underwent similar pathways) was offered to patients and families in order to give the possibility of a fellow patient giving him/her personal opinion about the current quality of life after the surgery.

All the surgeries were performed using a minimally invasive approach. The TME was performed using a synchronous abdominal and transanal approach (taTME). Intra- and postoperative outcome data were recorded, including operative time, conversion rates, length of stay (LOS), 30-day complications according to the Clavien-Dindo scale (19) and the need for re-operation or re-admission. The postoperative data consisted of a full pathological report with tumor regression grade including the according to Ryan and mismatch repair protein expression (20). Postoperative functional evaluation was carried out using fTRST, ADL and ECOG-PS.

### TNT protocols

TNT was offered to patients in case high-risk features were reported during the staging including at least one of the following: threatened circumferential resection margins (CRM+s), the presence of extramural vascular invasion (EMVI+), metastatic mesorectal and extramesorectal lymph nodes.

The TNT was defined as neoadjuvant chemotherapy (nCT) followed by short-course (5 × 5 Gy) or standard long-course radiotherapy (28 daily fractions of 1.8 Gy up to 50.4 Gy or 25 fractions of 2 Gy up to 50 Gy) with concurrent capecitabine (twice-daily oral capecitabine 825 mg/m^2^). Optional field reduction was recommended after 45 Gy (1.8 Gy schedule) or 46 Gy (2 Gy schedule), with the last fractions delivered to the tumor bed. The clinical target for radiotherapy (RT) included the entire mesorectum with the primary tumor and relevant regional lymph nodes. All the patients underwent RT starting 3 weeks from the end of the nCT.

The nCT regimen consisted of 4 cycles of CAPOX (capecitabine 1,000 mg/m^2^ orally twice daily on days 1–14, oxaliplatin 130 mg/m^2^ intravenously on day 1, and a chemotherapy-free interval between days 15–21) or 8 cycles of modified FOLFOX-6 (mFOLFOX-6) which consisted of oxaliplatin (85 mg/m^2^) given as a 2-h intravenous infusion, followed by leucovorin (400 mg/m^2^) given as a 2-h intravenous infusion, followed by fluorouracil (400 mg/m^2^) given as an intravenous bolus and then as continuous intravenous infusion (2,400 mg/m^2^) over 46 h every 14 days. The choice of CAPOX or mFOLFOX-6 was determined by the treating physician and according to hospital policy. In the case of toxicity, there was a dose reduction of 25% or more.

Multimodality treatment feasibility was assessed based on patient postoperative functional status, and evaluation of treatment completion and toxicity in addition to the need for a dose reduction. The chemotherapy dosing for the first cycle of treatment was categorized as standard or dose reduced as per the National Comprehensive Cancer Network (NCCN) guidelines (21). The patients were followed from the beginning until the end of the chemotherapy course. Toxicity was measured at each clinical encounter using the National Cancer Institute Common Terminology Criteria for Adverse Events (NCI CTCAE), version 5.0 (22).

### Statistical analysis

The continuous variables were measured using median, mean and range. The categorical variables were described by count and relative frequency. The data were analyzed using STATA 16.1 (StataCorp, College Station, Texas). The primary aim was to evaluate surgical and oncologic outcomes in older patients who underwent TNT for LARC. The secondary aim was to explore the feasibility of this emerging approach in this subset of patients.

## Results

From November 2017 to April 2022, a total of 15 patients were found to be eligible for this analysis. Baseline demographics, frailty screening and rectal cancer clinicopathological characteristics are summarized in Table 1. The mean age of patients was 74 (range 70–81) years; 8 were male (8/15, 53.3%). The median BMI was 26.2 (range 22–32) kg/m^2^. The mean distance of the tumor from the anal verge was 5.2 cm. During pre-treatment staging, 14 patients had positive nodes (14/15, 93.3%), 11 patients (11/15, 73.3%) showed involvement of the circumferential resection margin (CRM+) and EMVI+ was reported in 10 cases (10/15, 66.6%).

**Table 1:** Patient characteristics.

Age (median, range)	74 (70–81)
Gender: *n* (%)	
Male	8 (53.4%)
Female	7 (46.6%)
Body Mass Index (BMI): kg/m^2^ (median, range)	26.2 (22–32)
Charlson Comorbidity Index (CACI) (median, range)	5 (5–8)
Flemish Triage Risk (fTRST) (median, range)	1 (0–2)
Activities of Daily Living (ADL) (median, range)	6 (5–6)
Eastern Cooperative Oncology Group Performance Status (ECOG-PS) (median, range)	1 (0–2)
American Society of Anesthesiology (ASA) score (median, range)	3 (2–3)
Distance from the anal verge (mean, range)	5.2 (3–8)
T (*n*, %)	
2	0
3	13 (87%)
4	2 (13%)
N+ (*n*, %)	11, 93.3%
Threatened Circumferential Resection Margin (CRM+) (*n*, %)	10, 66.6%
Extramural Vascular Invasion (EMVI+) (*n*, %)	10, 66.6%

A frailty assessment was carried out at baseline (see Table 1). Ten patients (10/15, 66.6%) received mFOLFOX-6 and 5 CAPOX (5/15, 33.3%). Minimal toxicity was reported in both groups, necessitating a dose reduction during treatment. All the patients completed their neoadjuvant chemotherapy plan. Details regarding the treatment regimen, toxicity, and pre- and post-treatment staging are summarized in Table 2. Fourteen patients (14/15, 93.3%) underwent long-course RT with concurrent capecitabine whereas one patient underwent short-course treatment. The degree of toxicity was also acceptable during the radiation therapy with the exception of one patient (1/15, 6.6%) who could not complete the treatment due to diarrhea, dehydration, and acute kidney failure.

**Table 2:** Neoadjuvant treatment and restaging data.

Treatment regimen (*n*, %)	
mFOLFOX-6	10, 67%
CAPOX	5, 33%
Cycles (median, range)	5.4 (3–8)
Toxicity (*n*, %)	
G0	1, 6.6%
G1	7, 46.7%
G2	3, 20%
G3	3, 20%
G4	1, 6.6%
Radiotherapy (*n*, %)	
SCRT	1, 6.6%
LRT	14, 93.4%
Toxicity (*n*, %)	
G0	3, 20%
G1	6, 40%
G2	2, 13%
G3	3, 20%
G4	1, 7%
Radiological response (*n*, %)	
CR	0
PR	15, 100%
SD	0
PD	0
T (*n*, %)	
0	1, 7%
1	1, 7%
2	9, 60%
3	2, 13%
4	2, 13%
N+ (*n*, %)	4, 27%
Threatened Circumferential Resection Margin (CRM+) (*n*, %)	4, 27%
Extramural Vascular Invasion (EMVI+) (*n*, %)	1, 7%

All the patients showed radiological partial response (PR) after TNT, and no interval disease progression was recorded. At restaging after treatment, positive nodes were reported in 4 cases (4/15, 26.6%), the CRM+ was still described by the MRI in 4 cases (4/15, 26.6%), and EMVI+ in 1 patient (1/15, 6.6%).

Details regarding the surgical procedures are summarized in Table 3. All procedures were performed using a laparoscopic approach; no conversion to open surgery was recorded. The median operating time was 280 min (range 110–420). An abdominoperineal resection (APR) was performed in 7 cases (7/15, 47%), a TaTME with coloanal anastomosis and diverting loop ileostomy was performed in 7 patients (7/15, 47%). One patient (1/15, 6%) required an APR associated with a colpohysterectomy due to a locally advanced tumor. All the patients were treated according to the standard Enhanced Recovery After Surgery (ERAS) protocol. Median LOS was 4 days (range 3–29). Postoperative complications were minimal; however, 1 patient had a Clavien-Dindo grade 4 complication (anastomotic leak and acute cholecystitis which required reintervention and intensive care unit management). No readmission within 30 days from surgery was reported. No patient died within 90 days from surgery. A pathologic complete response (pCR) to TNT was reported in 3 cases (3/15, 20%). All the patients had R0 resections, regardless of their preoperative staging, with negative circumferential and distal resection margins at final pathology (see Table 4). No patients underwent adjuvant therapy, regardless of the tumor stage.

**Table 3:** Surgical details and postoperative outcomes.

Surgical procedure (*n*, %)	
Abdominoperineal resection (APR)	7, 47%
Low anterior rectal resection (LAR) + diverting ileostomy	7, 47%
LAR + diverting ileostomy + colpohysterectomy	1, 6%
Mean operative time (min, range)	280 (110–420)
Laparoscopy (*n*, %)	15, 100%
Length of Stay (LOS) (median, range)	4 (3–29)
Clavien-Dindo (*n*, %)	
1	1, 7%
2	5, 33%
3a	0
3b	0
4a	0
4b	1, 7%
5	0
No complications (*n*, %)	8, 53%
30-day reoperation (*n*, %)	1, 7%
30-day readmission (*n*, %)	0
30-day fTRST (median, range)	1 (0–2)
30-day ADL (median, range)	6 (5–6)
30-day ECOG-PS (median, range)	1 (0–2)

**Table 4:** Oncological outcomes.

ypT (*n*, %)	
0	3, 20%
1	1, 7%
2	6, 40%
3	2, 13%
4	3, 20%
ypN+ (*n*, %)	4, 27%
R0 (*n*, %)	15, 100%
Completeness of mesorectal fascia	15, 100%
Complete pathological response (ypT0N0) (*n*, %)	3, 20%
Harvested lymph nodes (mean, range)	21.5 (10–53)
Ryan tumor regression score (*n*, %)	
0	3, 20%
1	4, 27%
2	1, 7%
3	7, 46%
Mismatch repair protein expression (*n*, %)	
Normal	13, 87%
Deficient	2, 13%

When functional assessment was repeated, 30 days after surgical treatment, no differences were reported between before and after treatment (see Table 3).

## Discussion

The present study showed that TNT followed by TME was feasible and safe in fit older patients, having good short-term oncologic outcomes. The present results show optimal compliance to TNT, which was completed with acceptable toxicity in all cases. Regarding postoperative complications, only one patient had a Clavien-Dindo grade 4 complication, and neither reintervention nor deaths within 30 days after surgery were reported.

Age should not be the main determinant in the treatment decision-making process; individual patient frailty should be defined to promote optimal care tailored to each patient's specific needs (8). Older adults are a heterogeneous population covering a spectrum from fit to vulnerable to frail; therefore, individual evaluation of patient performance is the correct way of defining the feasible pathways for each patient. In the present series, patient evaluations were always presented during the MDT by a geriatrician or a frailty expert; these evaluations impacted the decision-making process, helping to define which patients should undergo full TNT.

The time spent assessing frailty was well spent as it was shown that older patients, selected based on their fitness level, could complete a multi-treatment maximally impactful pathway, such as TNT, for RC management. All the patients enrolled in the present study showed no differences in ADL, fTRST, and ECOG-PS score before and after completing treatment (medical and surgical) for LARC with limited toxicity and a low postoperative complication rate. Optimal cancer care, even when highly impactful such as in the case of TNT, should no longer be denied to older patients based on age alone.

The idea of TNT for LARC has been included as a pre-operative treatment option by the Clinical Practice Guidelines in Oncology (National Comprehensive Cancer Network, NCCN) since 2018, although only recently have the results of the RAPIDO and PRODIGE23 trials started to change clinical practice (16, 17). These two randomized clinical trials have addressed the effects of TNT for LARC over the standard neoadjuvant chemoradiotherapy (nCRT), adopting short-course radiotherapy (SC-RT) and induction nCT, respectively. In both studies, patients enrolled in the TNT arm showed a better pathologic complete response (pCR) rate and disease control, increased disease-free survival (DFS), and decreased disease-related treatment failure with better tolerance as compared to adjuvant therapy. Therefore, the TNT approach has gained interest as a possible means of improving long-term oncologic outcomes in patients with LARC.

The RAPIDO study, which is based on short-course radiation and systemic chemotherapy, experimental arm included 189 patients older than 65 (39% of the population). Unfortunately, not only 65 years seems to be a suboptimal cutoff to identify the older population, and a subgroup analysis is not provided in the publication. What has been shown instead, following the RAPIDO regimen, is that only 78% vs. 85% (standard arm) of patients underwent an optimal TME with intact mesorectum and that 1/10 patients had an R1 resection (16). Our much smaller experience shows in contrast that best surgical care can be achieved in a higher percentage of patients.

To date, no study specifically reporting on TNT in older patients has been published; however, many centers are also clearly incorporating this option in their armamentarium for treating this group. Moreover, this is happening without any specific oncologic trial being run on this group or the patient fitness level being assessed (in many cases) (23). Older patients are excluded based only on age alone or included without knowledge regarding their frailty (4). This has once again been shown by Aparicio et al. who analyzed 110 patients over 75 years of age who were managed for CRC and found that older patients received sub-standard treatment in 52% of cases. The majority underwent surgery whereas only a few underwent chemo- or radiotherapy (24).

Accurate diagnosis and pre-operative assessment are crucial for choosing the appropriate treatment, especially for older patients. Artificial intelligence (AI) is the new promising tool for achieving this goal. Machine learning (ML) is a field of AI that can develop mathematical algorithms capable of automatically learning different tasks with minimal human involvement. ML includes convolutional neural networks (CNNs), neural networks, and deep learning (DL). Both ML and DL have been massively adopted over these years to increase precision in diagnosis. Radiomics analyses quantitative data from diagnostic images that can be combined with ML to find other features beyond those obtained by radiologists. DL has been proven efficient in extracting information about polyp detection, cellular characteristics of RC, tumor environment and ratio, and estimating patient survival (25, 26). Regarding CT colonoscopy, further research is required to establish the role of DL in optimizing RC diagnosis, but DL could have a promising involvement in data extraction from CT scans which can increase the rate of diagnosing extraperitoneal CRC with a sensitivity of about 95% (27). However, MRI has been established as the most valuable imaging modality for primary staging and restaging after neoadjuvant treatment for RC and AI could improve accuracy in diagnosis and prognosis. Decision support tools based on MRI radiomics and ML have been developed to assist radiologists in differentiating parietal layers involved in the tumor and establish the T stage. AI has been proposed also as a tool to optimize the evaluation of lymph nodes (LNs) status. Recently, Ma et al. have been demonstrated that ML models could differentiate N0 from N1/N2 stages with a sensitivity and a specificity of 79% and 72%, respectively (28). Some authors also explored the possibility of predicting pCR through radiomics nomograms with promising results, even if it is not possible to draw a reliable conclusion (29, 30). An early and accurate diagnosis and prediction of neoadjuvant therapy response could significantly improve the management of patients with RC, mostly older patients who primarily benefit from the development of a tailored treatment.

In the present study, all patients were able to complete the entire neoadjuvant treatment pathway, although dose reduction was needed in the majority of cases. This was in contrast to what had previously been published by Margalit et al. who analyzed the rate of treatment deviation in 36 patients 75 years of age and older having rectal cancer treated with combined modality therapy. They reported an 83% rate of early treatment termination, interruption, or dose reduction. The rate of treatment deviation did not differ between patients who had preoperative or postoperative chemoradiotherapy (19% vs. 17%, *p* = 1.00); no significant differences in the deviation between patients with no to mild vs. moderate to severe comorbidities (*p* = 0.66) or no to moderate vs. severe comorbidities (*p* = 1.00) were reported (31).

The role of minimally invasive surgery (MIS), such as conventional or robotic-assisted, in reducing surgical trauma and improving postoperative recovery has been broadly discussed and analyzed for colorectal surgery; however, limited evidence is available regarding the effectiveness of the MIS approach in older patients with rectal cancer (32, 33). Several studies have demonstrated that age should not be considered the only significant predictor of postoperative morbidity and laparoscopic rectal cancer surgery can be safely performed, even in older patients (34, 35).

In the present study, all surgeries were performed using a conventional laparoscopic approach (namely taTME), and no conversion to an open approach was reported. The advantages of MIS become more important in geriatric patients who have limited resources for overcoming surgical stress when compared to younger counterparts. In the past, some authors argued that longer operating times and the need for the pneumoperitoneum and Trendelenburg position, which reduce venous return and potentially compromise ventilation, might significantly reduce the safety of the MIS approach in these patients (36); however, the above was not experiences in our series.

The majority of patients in our study underwent non-restorative procedures. In rectal cancer surgery, the choice between low anterior resection (LAR) with diverting loop ileostomy (DLI) and APR depends on the distance of the tumor from the anal verge/involvement of the sphincter complex, the technical feasibility of an anastomosis, the patient's clinical conditions and preferences, in light of the non-insignificant rate of suboptimal functional results after a colo-anal anastomosis (37).

In fact, besides clinical and surgical considerations, health-related quality of life (HRQoL) should be considered an important parameter of treatment efficacy. Data from the Dutch Colorectal Surgical Audit group about older patients who underwent low anterior resection (LAR) with diverting loop ileostomy (DLI) have shown that ileostomy closure was performed only in 68% of cases in the age range of 71–80, whereas only 60% of patients >81 years old had their stoma reversed. Moreover, DLI is a risk factor for readmission also after ileostomy closure (38)*.* The recently published GOSAFE study also showed that QoL is not improved, specifically in this group of patients, by a restorative procedure while complication rate (certainly higher after a colo-anal anastomosis as compared to an inter-sphincteric APR) clearly affects short and long-term QoL (39)*.* Indeed, several studies showed that older patients with permanent stoma have comparable HRQoL to older patients without a stoma or to normal population (40–42).

Balancing pros and cons of a coloanal anastomosis with the potential harm derived from postoperative complications might be difficult in older patients due to their limited physiologic reserves, therefore in these cases and inter-sphincteric APR seemed a viable option, after a thorough shared decision-making process. Appropriate counseling about patients' expectations and the potential side effects of a definitive DLI is advised in order to identify patients' real goals and understand which patients will be able to complete surgical treatment successfully (9).

In the present study, the median LOS was 4 days, and no readmission were reported. The present results may be biased due to a population of relatively fit patients with low frailty scores and an acceptable number of comorbidities. However, these findings point out the importance of careful patient selection based on performance scores, frailty assessment, and tumor characteristics. Moreover, the reduction in LOS due to MIS combined with enhanced recovery pathways improved postoperative functional recovery as reported in the present study group.

The vast majority of the patients underwent long-course RT (LCRT) as the treatment of choice at our center. Recent data about LR after TNT with short-course radiation therapy from the RAPIDO trial has shown an association with an increased risk of LRR and a higher rate of suboptimal TME surgery (16, 43, 44). None of this evidence was generated specifically on a study population focusing on older patients, which is the essence of the current work. In this series, the selection of a SCRT protocol was limited to 2 patients who had severe logistic limitations and couldn't achieve consistency with the 25-day long LCRT.

Early oncologic outcomes were also noted, with all the patients being able to have an R0 resection. Three patients (3/15; 20%) experienced a pCR while clear distal and circumferential margins were reported in all cases regardless of the initial staging of the cancer. Despite not being able to report on the long-term effects of TNT on disease-free and overall survival due to the limited duration of the follow up, this study showed that the rate of pCR and CRM positivity was similar/better to what has been previously reported on younger patients (45, 46). This was a promising early result for older patients affected by rectal cancer.

## Conclusions

Despite the relevant incidence of rectal cancer in the older population there are few studies focusing specifically on this group. The Authors' experience regarding maximizing cancer care for fit older patients showed that extended treatment was feasible, even in the geriatric population when properly screened for frailty with good perioperative, functional, and oncologic outcomes.

The hope is that, starting from limited observational experiences such as this, more space will be given to geriatric patients in larger, prospective, oncologic trials in order to identify proper care tailored to each patient's specific needs.

## Data Availability

The raw data supporting the conclusions of this article will be made available by the authors, without undue reservation.
